# Automated analysis of liver fat, muscle and adipose tissue distribution from CT suitable for large-scale studies

**DOI:** 10.1038/s41598-017-08925-8

**Published:** 2017-09-05

**Authors:** Joel Kullberg, Anders Hedström, John Brandberg, Robin Strand, Lars Johansson, Göran Bergström, Håkan Ahlström

**Affiliations:** 1 Department of Radiology, Uppsala University, Uppsala, Sweden; 2 Antaros Medical, BioVenture Hub, Mölndal, Sweden; 3 Department of Radiology, Sahlgrenska University Hospital, Gothenburg, Sweden; 4 Institute of Medicine, Sahlgrenska Academy at the University of Gothenburg, Gothenburg, Sweden

## Abstract

Computed Tomography (CT) allows detailed studies of body composition and its association with metabolic and cardiovascular disease. The purpose of this work was to develop and validate automated and manual image processing techniques for detailed and efficient analysis of body composition from CT data. The study comprised 107 subjects examined in the Swedish CArdioPulmonary BioImage Study (SCAPIS) using a 3-slice CT protocol covering liver, abdomen, and thighs. Algorithms were developed for automated assessment of liver attenuation, visceral (VAT) and subcutaneous (SAT) abdominal adipose tissue, thigh muscles, subcutaneous, subfascial (SFAT) and intermuscular adipose tissue. These were validated using manual reference measurements. SFAT was studied in selected subjects were the fascia lata could be visually identified (approx. 5%). In addition, precision of manual measurements of intra- (IPAT) and retroperitoneal adipose tissue (RPAT) and deep- and superficial SAT was evaluated using repeated measurements. Automated measurements correlated strongly to manual reference measurements. The SFAT depot showed the weakest correlation (r = 0.744). Automated VAT and SAT measurements were slightly, but significantly overestimated (≤4.6%, p ≤ 0.001). Manual segmentation of abdominal sub-depots showed high repeatability (CV ≤ 8.1%, r ≥ 0.930). We conclude that the low dose CT-scanning and automated analysis makes the setup suitable for large-scale studies.

## Introduction

Studies of body composition can improve the understanding of for example type-2 diabetes (T2D) and cardiovascular disease (CVD) and their prediction and prevention. Medical imaging techniques, such as Magnetic Resonance Imaging (MRI) and Computed Tomography (CT) can be used for detailed studies of human body composition. These techniques are often used to quantify liver fat, muscle, and adipose tissue distribution. Increased liver fat can be estimated from liver attenuation measured by CT and is believed to be crucial component in development of dyslipidemia associated with obesity. Abdominal adipose tissue is often separated into subcutaneous and intra-abdominal, or visceral adipose tissue, (SAT and VAT, respectively)^1–6^ as these are known to have different associations to glucose tolerance^7^ and thereby to T2D and CVD risk. More recently the importance of further separating these into sub-depots has gained attention. The VAT depot can be separated into intra- and retroperitoneal adipose tissue^8–10^ (IPAT and RPAT, respectively) and the SAT can be separated into deep and superficial depots (DSAT and SSAT, respectively). DSAT and SSAT have been found to differ in structure and function^5, 11, 12^, in association to insulin resistance^5, 12^, and in triglyceride saturation^12, 13^. Quantification of thigh composition, in terms of muscle and adipose tissue sub-depots has also been found of importance in metabolic studies^14–17^.

CT creates images in absolute Hounsfield units (HU) but exposes the subject to ionizing radiation limiting the number of slices that can be obtained. The Swedish CardioPulmonary bioImage Study (SCAPIS)^18^, has a primary aim to study cardiovascular and pulmonary disease for which CT angiography and CT of the lungs are preferred methods. The study also includes CT imaging of liver, abdominal adipose tissue and thighs for the purpose of detailed body composition phenotyping. It aims to include 30,000 study subjects in the age range 50 to 64 years in Sweden so parameters of high throughput and economy were also important for the selection of imaging modality. In 2012, a pilot study of 1111 subjects was undertaken. In such large studies, both limited radiation exposure and analysis automation are important for the feasibility of data collection and analysis. Automated methods also give objective results free from bias that may be introduced by human operators. Manual interaction might however still be needed where full automation is difficult to achieve, e.g. when separating IPAT-RPAT or SSAT-DSAT.

The purpose of this study was twofold. The first was to develop and validate automated image processing techniques for analysis of body composition from CT image data. These should be suitable for large-scale studies, like the SCAPIS study, in terms of both accuracy and time efficiency. The second was to evaluate manual segmentation of abdominal adipose tissue sub-depots, where fully automated analysis was found difficult to achieve.

## Methods

### Subjects and use of images

The SCAPIS^18^ pilot trial recruited 1111 subjects of which 1089 underwent CT imaging for determination of body composition. The study included a randomly selected sample from the population registry and included men and women aged between 50 and 64 years living in the city of Gothenburg, Sweden. The only exclusion criteria applied was inability to understand written and spoken Swedish. The main reason for the dropouts was unwillingness to participate in the CT part of the study because of the associated radiation dose. SCAPIS has been approved as a multicentre trial by the ethics committee at Umeå University and adheres to the Declaration of Helsinki. The methods applied were carried out in accordance with the relevant guidelines and regulations. Written informed consent was obtained from all subjects.

During the method development different subsets of these images were randomly selected and used (see details below). A subset of 50 randomly selected subjects (without any stratification, age 58.6 ± 4.1 years, BMI 28.0 ± 4.2 kg/m^2^, 24 females) was used for the evaluation of automated and manual measurements. The training and evaluation of the SFAT depot however required that the fascia lata between SAT and SFAT to be visually identifiable. All 1089 thigh images were therefore reviewed and 57 (age 58.4 ± 4.5 years, BMI, 27.5 ± 3.2 kg/m^2^, 40 females) were found to have a visible fascia. These images were randomized in two groups that were used for algorithm training (n = 28) and evaluation (n = 29), respectively. Note that this low prevalence of visible fascia lata in these images only limits the analysis of the SFAT depot and not the other thigh target measures.

### CT imaging protocol

This study included CT images from the SCAPIS pilot study^18^. All subjects in this study were scanned with a 3-slice-CT-protocol imaging liver, abdomen, and thighs. The subject preparation and scanning details have previously been described in detail^18^.

A 5mm axial liver image slice was reconstructed from the volumetric lung images that were acquired using spiral imaging. The slice was chosen to include the both liver lobes and the spleen. Body composition images were acquired as two sequential images with a slice thickness of 5 mm one at mid-thigh level and one at abdominal level. The thigh image was positioned at one half of the measured distance between the outer acetabular edge and the knee joint. The abdominal image was acquired in the level of the fourth lumbar vertebra (L4).

For feasibility the time of the day when the exams were performed was not fixed. To standardize the liver glycogen levels the participants were given a standardized meal (Modifast, Nutriton&Santé) calculated based on body mass index (BMI) two hours prior to CT examination. The CT system performance was evaluated using daily phantom scanning with recalibrations using water a phantom approximately weekly or when needed. The dose for the three slices is subject dependent but was estimated to be on average 0.245 mSv which can be compared to an approximate yearly total dose of 3 mSv for persons living in Sweden.

### Automated image analysis

Automated image analysis algorithms were developed for assessment of liver attenuation, areas of abdominal SAT and VAT and areas of thigh adipose tissue depots and muscle area and attenuation. Following two previous studies^15, 17^ the thigh adipose tissue was split into the three sub-depots SAT, subfascial (SFAT) and intermuscluar (IMAT) and the present study aimed to estimate these using a fully automated procedure. The algorithms are described in detail below.

Throughout this work, i.e. for both automated and manual segmentations, attenuations above −300 HU were used to separate body (and patient table) from air. The attenuation range −190 to −30 HU was used for adipose tissue, the range −29 to +151 HU for lean tissue, and attenuations above 400 HU for cortical bone. The automated algorithms were implemented in C++ in an in-house developed image analysis platform.

### Automated quantification of liver attenuation

**Figure 1:**
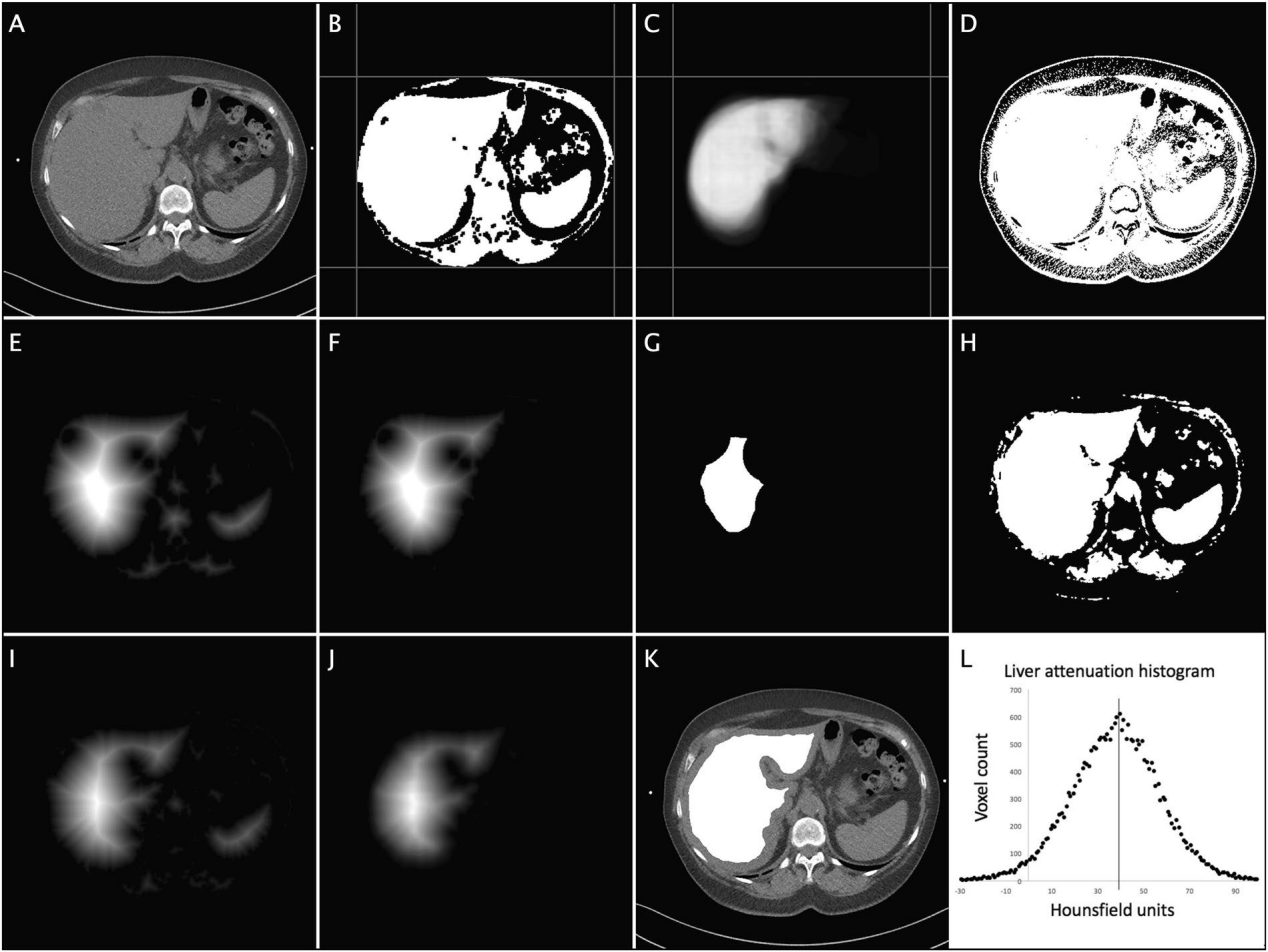
Illustration of the automated algorithm that determines liver attenuation. (**A**) the original CT image (Note the patient bed in the lower part of the image). (**B**) the result from the lean tissue segmentation (step 1) and the span of abdominal cavity, used to create and align the shape probability map. (**C**) the liver shape probability map (P_liver)_. (**D**) thresholded lean tissue from the original image. (**E**) distance transform (DT1) of thresholded image. (**F**) distance transform multiplied with probability map (DT1*P). (**G**) DT1*P thresholded that is used to estimate liver attenuation range (R). (**H**) original image thresholded using R. (**I**) distance transform (DT2) of the new thresholded image. (**J**) distance transform multiplied with probability map (DT2*P). (**K**) final segmentation superimposed on the original image. (**L**) Histogram with Gaussian fitting and a line indicating the center of the Gaussian.

Liver fat content was estimated by measurement of attenuation (HU) of liver tissue^19^. The liver tissue was segmented using an automated algorithm that achieved an approximate segmentation of the liver. The algorithm is described in detail in the algorithm outline and Fig. 1 below. A approximate segmentation was deemed acceptable, as the purpose was to estimate average tissue attenuation and not the liver size.

The liver segmentation use liver shape information from a set of reference segmentations as well as automatic subject-specific attenuation thresholding determined using attenuation sampling from the liver. The reference liver segmentations were performed in 25 randomly selected liver slices not included in the evaluation set. The derived shape information is combined into a probability map (P_liver_) by standardizing the geometries of the liver images. This was achieved by standardizing the span of the abdominal cavities (inside SAT). This span and the probability map are shown in Fig. 1B,C. The subject specific thresholding was applied since the liver attenuation was seen to vary widely between subjects complicating the segmentation of the liver.

### Automated quantification of abdominal adipose tissue

**Figure 2:**
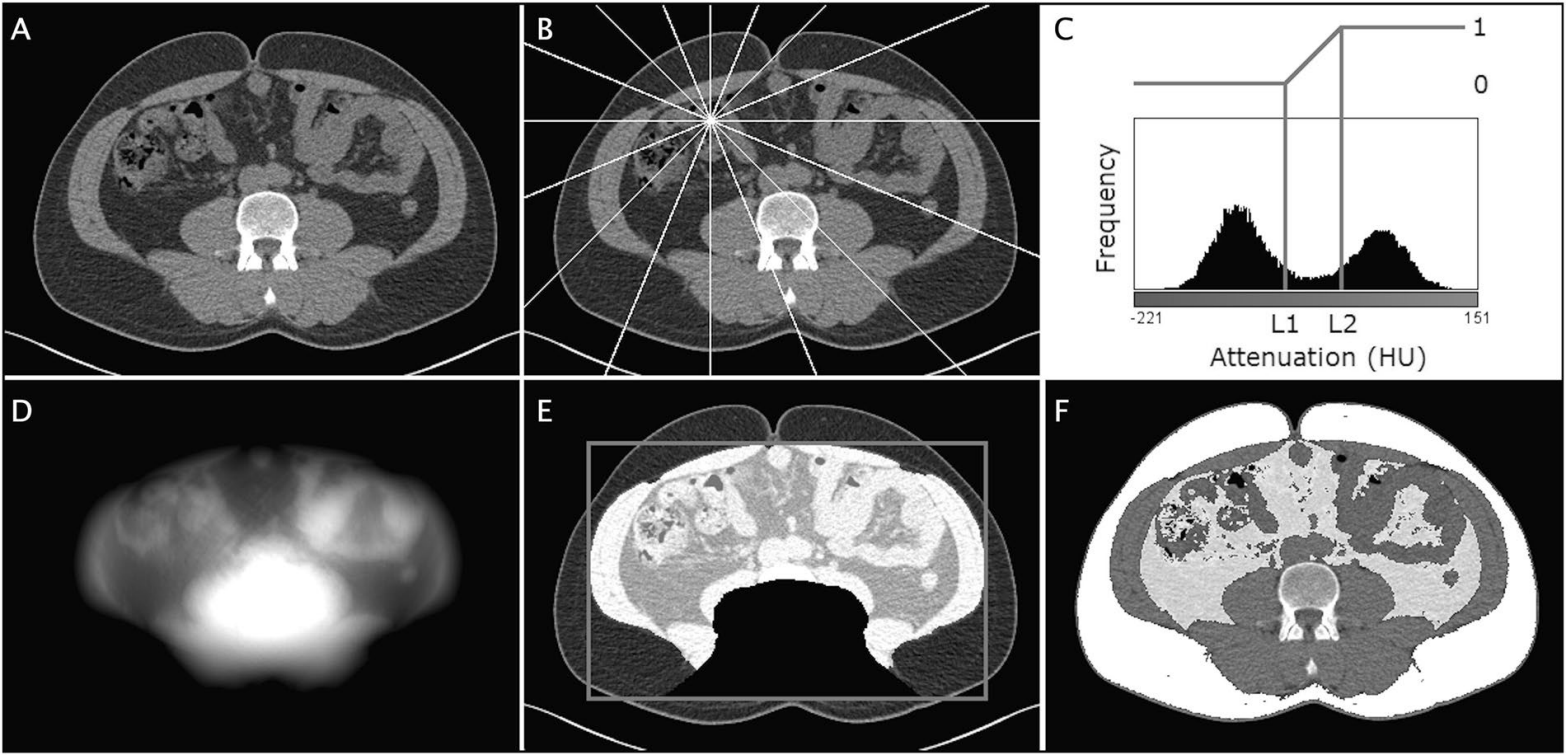
Illustration of the automated algorithm that segments VAT and SAT. (**A**) The original CT image. (**B**) How the inside lean tissue (ILT) filter “shoots rays” from each pixel in different directions. (**C**) Illustration of the mapping from attenuation to lean tissue probabilities, including the levels L1 and L2 that were used to define the linear ramp. (**D**) ILT filter response. (**E**) thresholded filter response superimposed on the original image. The image also shows the bounding box used to create and align the back probability map that masks the back region. (**F**) Automated VAT and SAT segmentation results superimposed on the original image.

The algorithm for segmentation of abdominal VAT and SAT has three main features, see algorithm outline below and Fig. 2. Firstly, adipose tissue is segmented using thresholding. Secondly a novel filter denoted inside lean tissue (ILT) filter is applied to separate VAT from SAT by identifying regions in the image that are inside lean tissue. Thirdly, adipose tissue close to the spinal column, i.e. inter and perimuscular^20^, is removed, as it neither is considered as VAT nor SAT. A heuristic approach was used to determine algorithm parameters using a set of ten randomly selected subjects that were not included in the evaluation cohort.

The response from the novel ILT filter can be thought of as a measure of how much lean tissue surrounds a given pixel. In more detail it is a composite measure of amount of lean tissue in different direction converted into a probability map (P_inside_). The estimation of the amount of lean tissue in different directions is determined by traversing the image in these directions and accumulating lean tissue probabilities. The number of directions used is denoted n_dir_ and the attenuation values are mapped into lean tissue probabilities using a linear ramp between two attenuation levels, L1 and L2, see Fig. 2C.

The conversion from accumulated lean tissue probabilities (A_lt-prob_) in different directions into a scalar filter response is performed by summing the smallest A_lt-prob_ –values in each pixel. The percentage of directions in which probability values are summed is denoted Dir_percent_. The probability map is normalized to the range 0 to 1.

The removal of back AT used a probability map (P_back_) indicating where back AT is likely to be located to exclude unwanted tissue from the VAT segmentation. This probability map was created using manual segmentation of this region in 50 randomly selected images not included in the evaluation. A bounding box of the abdominal cavity (located by the ILT filter) was used as a reference coordinate system to align the reference segmentations to each other and to align the probability map to new images, see Fig. 2E.

### Automated quantification of thigh composition

**Figure 3:**
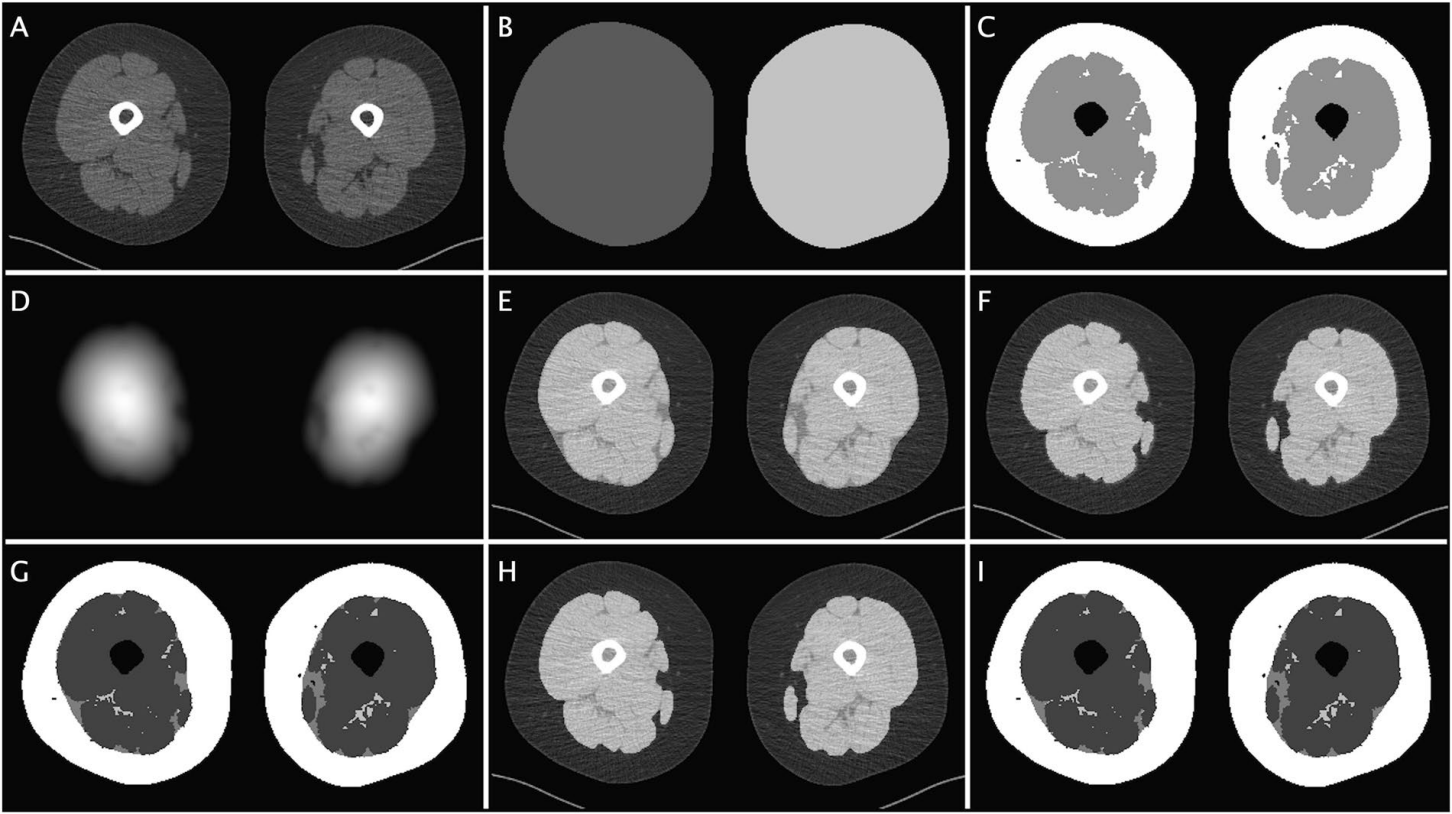
Illustration of the automated thigh segmentation algorithm. (**A**) The original CT image (note the patient table in the lower part of the image). (**B**) Segmented and separated thighs. (**C**) Separation of AT and muscle by thresholding and removal of bone. (**D**) The ILT filter response used to identify SAT. (**E**) The mask used to identify SAT overlayed on the original image. (**F**) The mask used to separate SFAT and IMAT from the ILT filtering approach (method 1). (**G**) The segmentation result where the method 1 is used to separate SFAT and IMAT. (**H**) The mask used to separate SFAT and IMAT from the morphological approach (method 2). (**I**) The segmentation result from method 2.

Fully automated methodology was developed for segmentation of muscle, SAT, SFAT, and IMAT from the thigh images, see algorithm outline and Fig. 3 below. The muscle segmentation was additionally used to estimate muscle attenuation^14, 15^. SAT and muscle was segmented using one method while two different methods were developed for the separation of the SFAT and IMAT depots. The results from these two methods will henceforth be referred to as SFAT1/SFAT2 and IMAT1/IMAT2, respectively. These methods are described below and in algorithm steps 4.1 and 4.2.

Firstly, the thighs were segmented and separated. The proceeding steps were performed on one thigh at a time. Secondly, muscle and AT were segmented by thresholding. The SAT depot was then identified using the ILT filtering technique. Next, the two different methods were used to segment SFAT and IMAT. The first method used the ILT filter technique and the other method used morphological operations. The parameters of both methods were determined using training on a set of manual reference segmentations primarily optimized adipose tissue area correlations and secondarily segmentation accuracy as measured by the Dice coefficient.

### Validation of the automated image processing techniques

**Figure 4:**
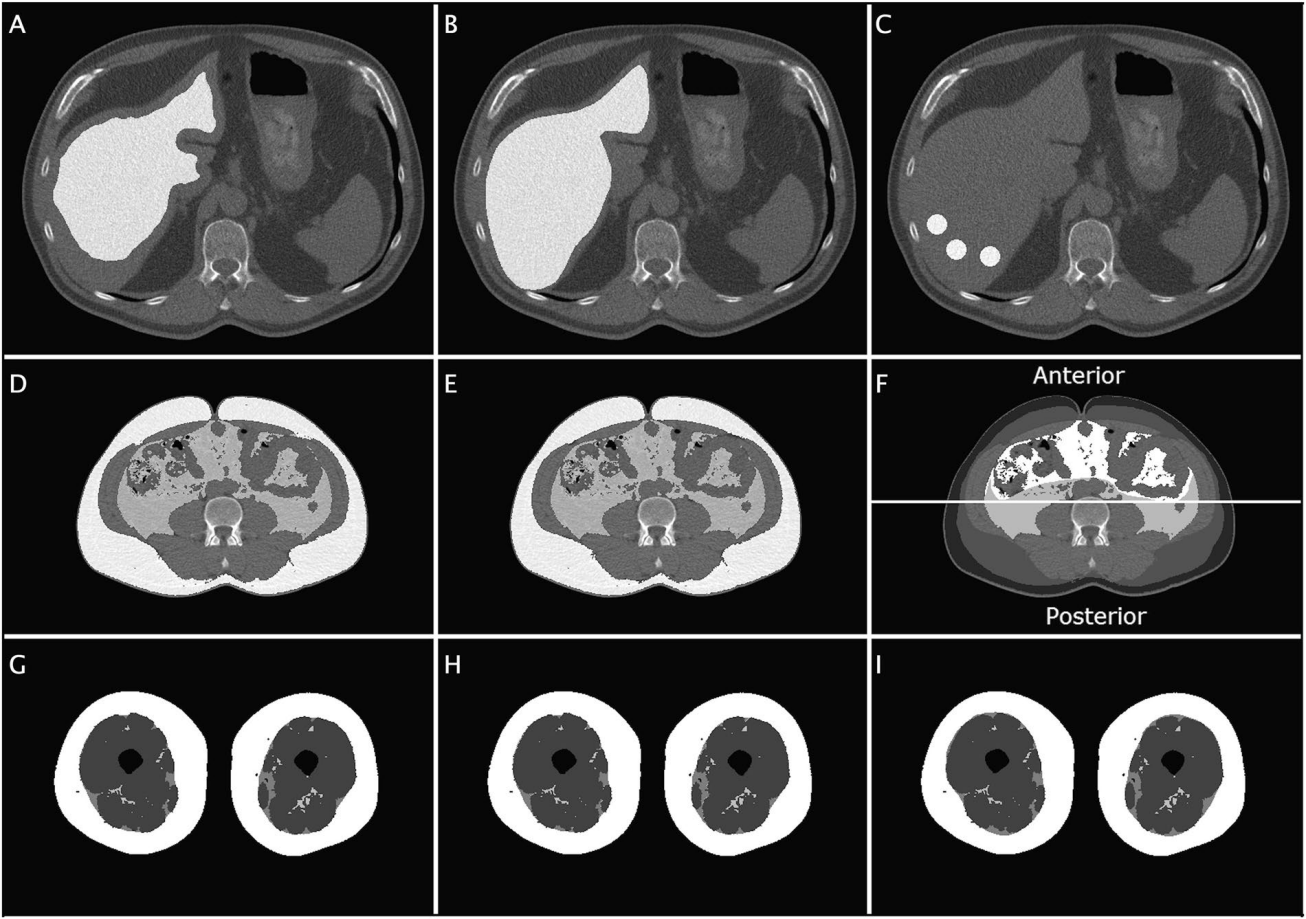
Illustration showing the different automated segmentation results and corresponding manual references. (**A**) Automated liver segmentation. (**B**) Manual delineation of the liver. (**C**) Manual identification of the dorsal ROIs. (**D**) Automated segmentation results of abdominal VAT and SAT. (**E**) Manual abdominal reference segmentation. (**F**) Manual delineation of the abdominal subdepots, SSAT, DSAT, RPAT, IPAT. (**G**) Automated thigh segmentation results from method 1. (**H**) Automated thigh segmentation results from method 2. (**I**) Manual reference segmentation of the thighs.

The liver attenuation was validated using two different manual protocols, see Fig. 4B,C. The first protocol (Manual 1, operator M.A.) segmented the liver just inside the liver border to avoid partial volume effects. The second protocol (Manual 2, operator M.K.) used manual placement of three small ROIs in the dorsal part of the liver. In Manual 1, the liver attenuation was determined using the same histogram-fitting technique as in the automated analysis. In Manual 2, the average value of the three mean ROI values was used as the estimate of liver attenuation.

The automated VAT and SAT segmentations were validated using manual segmentations as reference, see Fig. 4E. The manual segmentation comprised delineation of two contours. The first was the inner boundary of the abdominal muscle wall and the second was the outer boundary of the muscle wall. All adipose tissue inside the first contour, and outside the second contour was used as reference VAT and SAT segmentations, respectively.

The automated measurements of muscle areas were validated using manual segmentation and thresholding of lean tissue using the range in HU for lean tissue.

The SAT, SFAT and IMAT segmentations from the thighs were validated using the manually segmented 29 images with visible fascia that were not used in the method development, see Fig. 4I.

The automated assessment of muscle attenuation was compared to two manual protocols. The first (Manual 1) consisting of manual delineation of two large muscle ROIs, one in each leg, avoiding IMAT. The second (Manual 2) used a manual positioning of two elliptical ROIs in muscle tissue, one in each leg, avoiding IMAT. The average value of the two measurements was used.

The execution times for the automated assessments were measured when executed on a computer with an Intel Core I7 3.4 GHz CPU with 16GB ram. Manual segmentation were performed using the software ImageJ (version 1.42q).

### Manual assessment of abdominal subdepots and their evaluation

Manual segmentations of IPAT, RPAT, DSAT, and SSAT were performed as the importance of these depots is going to be studied in the SCAPIS project and as they are very difficult to assess using automation from these images. The manual segmentations were therefore evaluated using repeated measurements in the same randomly selected 50 subjects specified above. The same operator (M.A.) performed the segmentations twice approximately one month apart.

IPAT/RPAT was measured using a scheme developed together with two experienced radiologists (H.A., J.B.), see Fig. 4F. The segmentations were performed by separating the previously described manual VAT segmentation in two subdepots. This was performed by delineating the contour of the RPAT depot. The delineated contour was drawn in muscles and through intra-abdominal adipose tissue. The line through adipose tissue was drawn posterior to ascending and descending colon and small intestine and anterior to the great vessels.

DSAT and SSAT were quantified by delineation of Scarpas fascia where it could be seen. The automated SAT segmentation was split in two using this delineation. Since the fascia could not always be seen around the entire abdomen a system for splitting anterior and posterior regions was used in the proceeding analysis, see Fig. 4F. The line separating anterior and posterior was positioned automatically in the center of gravity of the segmented abdomen. DSAT and SSAT were extracted anterior and posterior to this line only if the fascia was delineated entirely in respective half.

### Statistics

Two-tailed paired t-test and linear (Pearson) correlations were performed. Dice coefficients, that measure the degree of overlap between two segmentations, were calculated as: Dice = 2*TP/(2*TP + FP + FN), where TP, FP, and FN are the number of true positive, false positive, and false negative pixels, respectively. Statistical evaluations were carried out using Statistica (Version 12, Statsoft, Inc). P-values < 0.05 were considered statistically significant.

## Results

**Figure 5:**
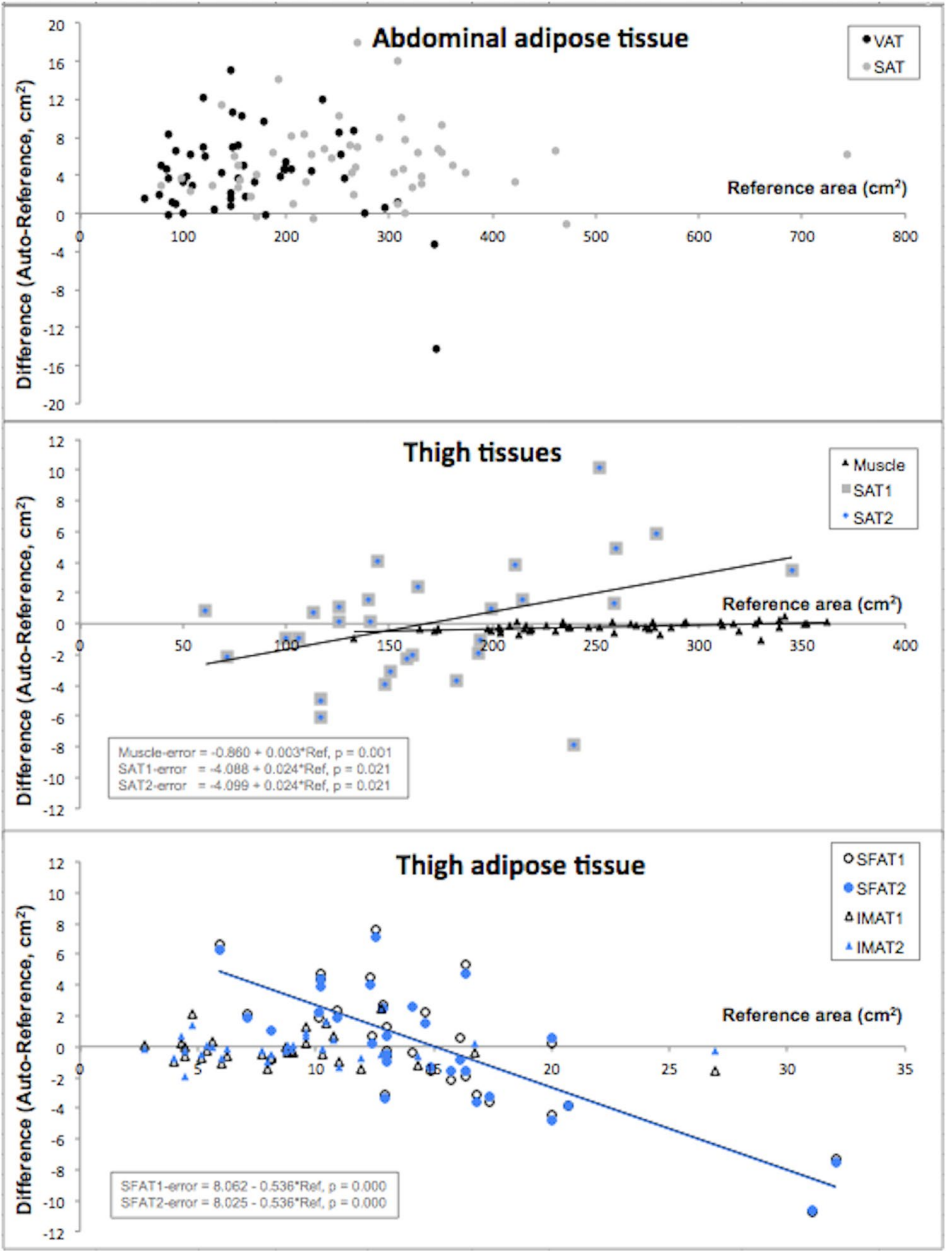
Plots of method evaluations and comparisons. The plots show measurement error on the y-axis and reference or manual measurement on the x-axis. Blue colour is used for method 2. (**A**) Visceral adipose tissue (VAT) and subcutaneous adipose tissue (SAT) areas. (**B**) Thigh subcutaneous adipose tissue (SAT, method 1/2) and muscle areas. (**C**) Thigh subfascial adipose tissue (SFAT, method 1/2) and intermuscluar adipose tissue (IMAT, method 1/2) areas.

**Figure 6:**
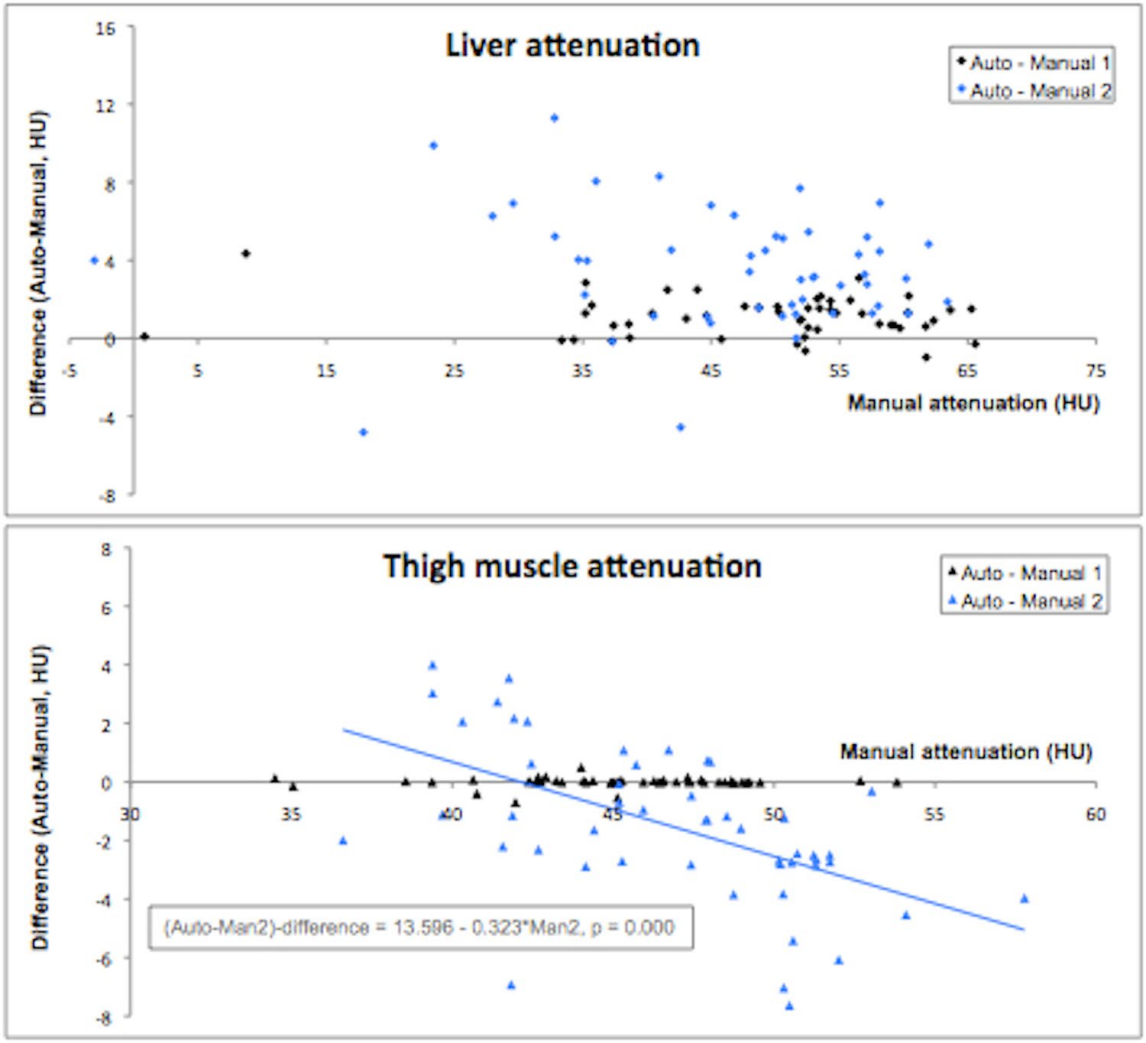
Plots of method evaluations and comparisons. The plots show measurement error on the y-axis and reference or manual measurement on the x-axis. Blue colour is used for method 2. (**A**) Liver attenuation: Automated compared to Manual 1 and Manual 2. (**B**) Muscle attenuation: Automated compared to Manual 1 and Manual 2. Manual 1: Manual segmentation of the majority of the liver/muscles, respectively. Manual 2: Manual segmentation of three dorsal liver ROIs and one elliptical muscle ROI, per leg, respectively.

The developed methods were successfully applied to all images during the evaluation. The evaluation results of area measurements are shown in Table 1 and Fig. 5, respectively and those for attenuation measurements in Table 2 and Fig. 6, respectively. The average execution times for the automated assessments were 13, 18 and 12 seconds for the liver, abdomen and thigh segmentations, respectively. Manual segmentation requires approximately 40 s for delineation of each region of interest, including image loading and saving of results.

**Table 1:** Results from the automated and manual assessments of areas and their validation.

	Area (cm^2^)	Area (cm^2^)	P-value	R-value	FP	FN	Dice
	Auto	Reference					
VAT	168.7 ± 71.8	164.5 ± 72.8	< 0.001	0.998	0.046	0.014	0.971 ± 0.01
SAT	272.1 ± 114.1	266.6 ± 113.8	< 0.001	0.999	0.026	0.003	0.986 ± 0.01
TAT	440.8 ± 147.4	431.1 ± 146.6	< 0.001	0.999	0.030	0.006	0.982 ± 0.01
	Measurement 1	Measurement 2					
IPAT	99.8 ± 45.7	96.2 ± 46.2	0.002	0.985	—		0.887 ± 0.04
RPAT	68.9 ± 29.1	72.5 ± 29.8	0.002	0.965	—		0.851 ± 0.06
DSAT-anterior*	39.8 ± 15.6	39.3 ± 15.6	0.684	0.930	—		0.880 ± 0.05
DSAT-posterior**	97.5 ± 36.0	97.9 ± 36.3	0.499	0.992	—		0.956 ± 0.02
DSAT-ant + post*	139.5 ± 47.7	139.1 ± 48.1	0.776	0.987	—		0.935 ± 0.02
SSAT-anterior*	53.0 ± 23.7	53.5 ± 22.6	0.684	0.969	—		0.910 ± 0.04
SSAT-posterior**	69.0 ± 28.7	68.5 ± 28.0	0.500	0.987	—		0.935 ± 0.03
SSAT-ant + post*	125.9 ± 46.6	126.3 ± 43.3	0.776	0.988	—		0.927 ± 0.03
	Auto	Reference					
Muscle (thigh)	257.1 ± 57.5	257.3 ± 57.4	< 0.001	1.000	0.002	0.001	0.998 ± 0.00
SAT1 (thigh)^†^	171.5 ± 68.0	171.4 ± 66.3	0.904	0.999	0.029	0.031	0.970 ± 0.01
SAT2 (thigh)^†^	171.5 ± 68.0	171.4 ± 66.3	0.911	0.999	0.028	0.031	0.970 ± 0.01
SFAT1^†^	15.0 ± 3.8	14.9 ± 5.9	0.896	0.720	0.426	0.344	0.636 ± 0.07
SFAT2^†^	14.9 ± 3.6	14.9 ± 5.9	0.940	0.747	0.420	0.341	0.640 ± 0.07
IMAT1^†^	8.7 ± 4.7	8.9 ± 4.8	0.325	0.978	0.110	0.131	0.881 ± 0.05
IMAT2^†^	8.7 ± 4.9	8.9 ± 4.8	0.341	0.988	0.106	0.128	0.881 ± 0.06

The number of subjects is 50 where not specified otherwise. *n = 31, **n = 47, ^†^n = 29.

Auto/Meas 1 is the automatic measurement for all results except for IPAT, RPAT, DSAT, and SSAT where they are the first of the two manual segmentations.

Manual/Meas 2 is the manual reference segmentation except for IPAT, RPAT, DSAT, and SSAT where they are the second manual segmentation.

The P-values correspond to the paired t-test performed in the measurement comparison.

TP/FP are the true and false positive ratios of the reference segmentations, respectively.

VAT – Visceral adipose tissue. SAT – Subcutaneous adipose tissue. IPAT – Intraperitoneal adipose tissue.

RPAT – Retroperetoneal adipose tissue. DSAT/SSAT – Deep/Superficial SAT, respectively.

SFAT1/2 – Subfascial adipose tissue from methods 1 or 2.

IMAT1/2 – Intermuscular adipose tissue from methods 1 or 2.

**Table 2:** Results from the automated and manual measurements of attenuation in the evaluation cohort (n = 50).

Attenuation (HU)	Auto	Manual 1	Manual 2	Correlations
Liver	49.9 ± 12.7*	48.8 ± 12.8*	46.3 ± 12.7*	0.997 / 0.971
Muscle (thigh)	44.9 ± 3.8*	44.7 ± 3.8*	46.8 ± 4.6*	0.999 / 0.835

Auto: Automated quantification of liver/muscle attenuation, respectively.

Manual 1: Manual segmentation of the majority of the liver/muscles, respectively.

Manual 2: Manual segmentation of three dorsal liver ROIs and one elliptical muscle ROI, per leg, respectively.

Correlations: Linear correlation coefficient (R) between automated and manual measurements.

*p-value lower than 0.001 between all three measurements.

### Abdominal adipose tissue

Automated measurements of VAT, SAT, and TAT showed very high correlations to the manual reference segmentations. However, the areas were significantly overestimated by the automated method. The overestimations were on average 4.6, 2.6, and 3.0%, respectively. The absolute errors for VAT and SAT did not show any association to the reference measurements, see Fig. 5A.

IPAT and RPAT showed high correlations between the first and second manual measurements. However, the mean values differed significantly. A 3.6% smaller IPAT depot was delineated during the second measurement. This corresponded to a 5.2% larger RPAT. The CVs for the repeated measurements were 5.2% and 6.4%, respectively.

The repeated measurements of DSAT and SSAT correlated less strongly in the anterior than in the posterior depots. The repeated measurements did not differ significantly. The CVs for DSAT and SSAT were 8.1%, 2.6%, 3.2% (anterior, posterior, combined) and 6.3%, 3.8%, 3.8%, respectively.

### Thigh composition

From the automated quantification of thigh composition, the muscle areas were the only component that differed significantly from the manual reference. The automated results were underestimated by on average 0.13%. This, in terms of biology, small, but relatively consequent pair-wise difference rendered a significant difference between the two techniques. The absolute errors in muscle, SAT1, SAT2, SFAT1, and SFAT2 areas were associated with the reference measurements, see Fig. 5B,C. The automated quantification of the SFAT depot showed the weakest correlation to the reference measurements. The second automated method that used a morphological approach tended to give the highest correlations to the manual reference segmentations. The differences was however not significant.

### Liver and thigh muscle attenuation

The automatic liver attenuation measurements showed higher correlation to the first manual method than to the second. The automatic results were significantly overestimated compared to both manual methods. The overestimations were on average 1.1 HU and 3.6 HU for Manual 1 and 2, respectively. No linear association was found between the difference between automated and the manual methods, see Fig. 6A.

The automatic thigh muscle attenuation measurements gave different results when compared against the two manual measurements. The automatic measurements were strongly correlated to the first manual method while a much weaker correlation was found to the second method (measured the mean attenuation in the two elliptical sub regions). The difference between the automated and the second manual method was also found associated to the measurements from this manual method, see Fig. 6B.

## Discussion

This work demonstrates fully automated segmentation methods for assessment of various liver, abdominal and thigh fat and muscle compartments from CT images. The use of only three CT slices limits the radiation exposure and the fully automated processing makes this setup suitable for large-scale epidemiological studies.

The algorithms presented contain basic morphological operations, shape prior information from manual segmentations, and a new type of filtering approach (ILT) that to the best our knowledge has not been presented previously. The basic idea of the filter is to examine the signal in different directions from a pixel of interest. In this application, lean tissue signal. This has the potential to sum signals from different directions to improve robustness to noise. The filter response also includes spatial information of this signal, here if the pixel in question is “inside”, i.e., surrounded by, lean tissue. In this work the ILT filter response was created by summing the directions with smallest accumulated probabilities. The result was then thresholded and used to separate the depots of interest. One might also use the rich information available in the accumulated lean tissue probabilities from different directions using other methods to generate segmentations. It has previously been described how intensity profiles along rays have been used to estimate local scale via identification of edges along the rays^21^ but to the best of our knowledge, this has not been used for segmentation.

In the abdominal images, the ILT filter helps the identification of the abdominal muscle wall, which is the key feature separating VAT and SAT. The idea behind the filter is the fact that pixels in SAT typically have much less lean tissue in approximately half of the probed directions. Intuitively, the combination of Dir_percent_ and the PM_threshold determines the convexity of the identified shape. A common problem in VAT/SAT separation is that subjects might have very thin muscles separating the depots. This is for example the reason why the test for discontinuities in previous work was needed^22^. One advantage of the ILT filter is that it relies on non-binary information from multiple directions and thus has the potential of being more robust.

Similar measurements have been performed in previous studies, most of which are smaller than the SCAPIS study and therefore manual or semi-automated approaches have been sufficient. Automated segmentation methods for assessment of liver^23^ and abdomen^22, 24, 25^ from CT image data have been presented earlier. Leg fat and muscle have previously been segmented from MRI data using automation^4, 26^. The present study is the first that presents an automated solution to the assessment of all three of liver, abdomen, and leg CT scans that is applicable to large-scale studies.

In general images were of high quality. None of the 50 VAT/SAT segmentations had to be excluded because of artifacts in the images affecting the results of the segmentation. The FOV of the abdominal images was also sufficiently large and did not truncate any parts of the abdomen.

Liver attenuation is of importance as it gives an indication of the chemical composition of the liver, such as its fat content. The automated segmentation of the liver was found complicated by the facts that liver attenuation typically overlapped those from other tissues and organs and that it was seen to vary largely (in this study in the range of 0-60 HU). In this work, the liver attenuation statistics was first sampled using an approximate segmentation and then used in the proceeding steps. A limitation by using thresholding based on sampled attenuations is that it might bias the measurements if the liver attenuation is very heterogeneous. To our experience, this is however not a common finding and none of the liver images analysed in this study showed a heterogeneity that could bias the results. A previous study has however measured the prevalence of focal fatty liver infiltration to 3% in healthy adults^27^. This might motivate a visual screening and eventual corrections for bias when applied in large-scale studies.

The automated results showed good correlation with manual measurements, but measured slightly higher attenuations than both manual segmentation methods (+1.1 HU and +3.6 HU). The second manual protocol (“Manual 2”) gave the lowest attenuation measurements. This was an expected finding as to our experience the dorsal part of the right liver lobe typically shows a lower attenuation than the rest of the liver. The differences measured depend on two factors; the region used for the measurement and the statistical processing, i.e. mean value or histogram function fitting. Since the first manual approach also used the histogram the only difference between this and the automated method was the liver region analysed.

Both automated VAT and SAT segmentations showed a high overlap with manual segmentations. In line with previous reports the automated SAT segmentation was found to agree better with the reference than the VAT segmentation^1, 2, 25^. As the overestimations of VAT and SAT areas by the automated method was not associated to the reference segmentations one might simply subtract this measured difference from future automated results if measurements more similar to the reference areas are sought. A limitation with the VAT segmentation is that it assumes that all adipose tissue inside the abdominal cavity outside the back mask is VAT, this is not fully correct as neither fat in the intestines nor adipose tissue in the abdominal wall is VAT. Previous studies have handled these sources of bias differently^22, 24, 25^.

Both VAT and SAT were overestimated compared to the reference segmentations. For VAT this was visually determined to be caused by inclusion of inter-muscular AT in the muscle wall and in the back muscles. The overestimation of SAT was seen to be mainly due to small leakage into the anterior part of the VAT depot. The automated method was seen to give a larger underestimation in one subject (−14.2 cm^2^ or −4.1%). This subject was seen to have a large VAT depot and relatively little muscles. The underestimation was seen caused by some leakage in the anterior muscle wall and some overestimation of back muscle by the back model. If deemed necessary a visual quality control of the resulting segmentations followed by eventual manual correction can be applied.

The separation of VAT and SAT into their sub-depots were performed manually in this study and this segmentation was evaluated using repeated measurements. This separation makes the evaluation results strongly dependent, as e.g. VAT is the sum of IPAT and RPAT. The IPAT and RPAT depots were separated using a manual segmentation protocol based on anatomical prior knowledge and DSAT and SSAT were separated where and when the fascia separating the two was visible. Despite the difficulties of manually segmenting the areas, the overlap between the two measurements were still fairly high (Dice ≥ 0.851). The mean values from the IPAT and RPAT found to differ significantly between the repeated measurements. This indicates that the operator likely changed the interpretation of the protocol slightly during this work. Even though the difference was rather small this type of error should be kept as low as possible through e.g. training or by averaging results from multiple operators and/or repeated measurements.

Both muscle, SAT and IMAT areas correlated very well with manual segmentations but the correlations for SFAT were significantly weaker (p < 0.001). The poor segmentation results for SFAT were mainly attributed to the fact that the fascia lata separating SAT and SFAT is not visible in the majority of these images. Hence the automated segmentation method could not be based on localization of this fascia but rather be trained to perform segmentation as similar as possible to the reference SFAT without explicit detection of the facia. The second automated method that used the morphological approach, tended to achieve slightly higher correlations. The fact that the fascia lata could only be visually identified in approximately 5% of the images collected in this study and therefore could only be evaluated in this subset, limits the extrapolation of the evaluation to the rest of the image data and study subject.

In conclusion, automated and manual methods for detailed analysis of body composition from a 3-slice-CT-protocol have been developed and evaluated. The use of low dose CT-scanning and computer aided analysis makes the setup suitable for large-scale studies. However, we have identified some limitations to this analysis, for example for the SFAT analysis, that should be kept in mind.

## References

[1] Positano V (2004). An accurate and robust method for unsupervised assessment of abdominal fat by MRI. J. Magn. Reson. Imaging.

[2] Kullberg J, Ahlström H, Johansson L, Frimmel H (2007). Automated and reproducible segmentation of visceral and subcutaneous adipose tissue from abdominal MRI. Int. J. Obes. (Lond)..

[3] Schwenzer, N. F. Quantitative Analysis of Adipose Tissue in Single Transverse Slices for Estimation of Volumes of Relevant Fat Tissue Compartments. *Invest. Radiol*. **45**, (2010). 10.1097/RLI.0b013e3181f10fe1 20829704

[4] Wald D (2012). Automatic quantification of subcutaneous and visceral adipose tissue from whole-body magnetic resonance images suitable for large cohort studies. J. Magn. Reson. Imaging.

[5] Kelley DE, Thaete FL, Troost F, Huwe T, Goodpaster BH (2000). Subdivisions of subcutaneous abdominal adipose tissue and insulin resistance. Am. J. Physiol. Endocrinol. Metab..

[6] Graffy, P. M. & Pickhardt, P. J. Quantification of Hepatic and Visceral Fat by CT and MR Imaging: Relevance to the Obesity Epidemic, Metabolic Syndrome, and NAFLD. *Br. J. Radiol*. 20151024, doi:10.1259/bjr.20151024 (2016). 10.1259/bjr.20151024 PMC5258166 26876880

[7] Sparrow D, Borkan GA, Gerzof SG, Wisniewski C, Silbert CK (1986). Relationship of Fat Distribution to Glucose Tolerance Results of Computed Tomography in Male Participants of the Normative Aging Study. Diabetes.

[8] Mantatzis M (2014). Abdominal adipose tissue distribution on MRI and diabetes. Acad. Radiol..

[9] Ross R, Aru J, Freeman J, Hudson R, Janssen I (2002). Abdominal adiposity and insulin resistance in obese men. Am. J. Physiol. Endocrinol. Metab..

[10] Koska J (2008). Distribution of subcutaneous fat predicts insulin action in obesity in sex-specific manner. Obesity (Silver Spring)..

[11] Sniderman AD, Bhopal R, Prabhakaran D, Sarrafzadegan N, Tchernof A (2007). Why might South Asians be so susceptible to central obesity and its atherogenic consequences? The adipose tissue overflow hypothesis. Int. J. Epidemiol..

[12] Marinou K (2014). Structural and functional properties of deep abdominal subcutaneous adipose tissue explain its association with insulin resistance and cardiovascular risk in men. Diabetes Care.

[13] Lundbom J, Hakkarainen a, Lundbom N, Taskinen M-R (2013). Deep subcutaneous adipose tissue is more saturated than superficial subcutaneous adipose tissue. Int. J. Obes. (Lond)..

[14] Goodpaster BH, Thaete FL, Simoneau Ja, Kelley DE (1997). Subcutaneous abdominal fat and thigh muscle composition predict insulin sensitivity independently of visceral fat. Diabetes.

[15] Goodpaster BH, Thaete FL, Kelley DE (2000). Thigh adipose tissue distribution is associated with insulin resistance in obesity and in type 2 diabetes mellitus. Am. J. Clin. Nutr..

[16] Livingston EH (2006). Lower body subcutaneous fat accumulation and diabetes mellitus risk. Surg. Obes. Relat. Dis..

[17] Rocha PM (2011). Visceral abdominal and subfascial femoral adipose tissue have opposite associations with liver fat in overweight and obese premenopausal caucasian women. J Lipids.

[18] Bergström G (2015). The Swedish CArdioPulmonary BioImage Study: Objectives and design. J. Intern. Med..

[19] Ducommun J-C, Goldberg HI, Korobkin M, Moss AA, Kressel HY (1979). The Relation of Liver Fat to Computed Tomography Numbers: A Preliminary Experimental Study in Rabbits. Radiology.

[20] Shen W (2003). Adipose tissue quantification by imaging methods: a proposed classification. Obes. Res..

[21] Saha PK (2005). Tensor scale: A local morphometric parameter with applications to computer vision and image processing. Comput. Vis. Image Underst..

[22] Zhao B (2006). Automated quantification of body fat distribution on volumetric computed tomography. J. Comput. Assist. Tomogr..

[23] Heimann T (2009). Comparison and evaluation of methods for liver segmentation from CT datasets. IEEE Trans. Med. Imaging.

[24] Chung, H., Cobzas, D., Birdsell, L., Lieffers, J. & Baracos, V. Automated segmentation of muscle and adipose tissue on CT images for human body composition analysis. 72610K, doi:10.1117/12.812412 (2009).

[25] Makrogiannis S, Caturegli G, Davatzikos C, Ferrucci L (2013). Computer-aided assessment of regional abdominal fat with food residue removal in CT. Acad. Radiol..

[26] Positano V (2009). Accurate segmentation of subcutaneous and intermuscular adipose tissue from MR images of the thigh. J. Magn. Reson. Imaging.

[27] El-Hassan AY, Ibrahim EM, Al-Mulhim FA, Nabhan AA, Chammas MY (1992). Fatty infiltration of the liver: Analysis of prevalence, radiological and clinical features and influence on patient management. Br. J. Radiol..

